# Socioeconomic status and lifestyle factors modifies the association between snack foods intake and incidence of metabolic syndrome

**DOI:** 10.1186/s12937-021-00728-y

**Published:** 2021-07-22

**Authors:** Parvin Mirmiran, Maryam Aghayan, Bahar Bakhshi, Somayeh Hosseinpour-Niazi, Fereidoun Azizi

**Affiliations:** 1 Department of Clinical Nutrition and Dietetics, Faculty of Nutrition Sciences and Food Technology, National Nutrition and Food Technology Research Institute, Shahid Beheshti University of Medical Sciences, Tehran, Iran; 2 Nutrition and Endocrine Research Center, Research Institute for Endocrine Sciences, Shahid Beheshti University of Medical Sciences, No 24, A’rabi St, Yeman Av, Velenjak, P.O.Box: 19395-4763, Tehran, Iran; 3 Endocrine Research Center, Research Institute for Endocrine Sciences, Shahid Beheshti University of Medical Sciences, Tehran, Iran

**Keywords:** Snack, Candies and chocolate, Metabolic syndrome, Socio-economic status, Adults

## Abstract

**Background:**

Intake of snack foods has been previously associated with an elevated risk of chronic disease; however, studies on snack foods and metabolic syndrome (MetS) while considering the modifying effect of socioeconomic status (SES) and lifestyle factors on this association are lacking. We aimed to investigate the association between snack foods intake and the MetS risk, and the mediatory effects of SES and lifestyle factors on the forenamed association among adults who participated in the Tehran Lipid and Glucose Study (2006–2018).

**Methods:**

This is a prospective study of 1915 participants (male, 40.5%), aged 19–74 year who were free of MetS at baseline. Dietary intakes were gathered using a validated, semi-quantitative food frequency questionnaire at baseline (2006–2008), and with 3-year intervals afterwards. Alternative approach was used for snack foods from all available questionnaires during follow-up. Snack foods were divided into 4 categories, including total snacks, biscuits and cakes, candies and chocolate, and salty snacks. Total snack foods intake and its subgroup (serving/week) were modeled as tertiles. MetS was diagnosed according to the Joint Interim Statement criteria. Physical activity level (PAL) categorized as low/medium and high levels. Information regarding smoking (Smoker/Non-smoker), education (higher/lower education), and occupation (employed/non-employed) was gathered using questionnaire. The Cox regression was used, regarding interaction between snack foods, SES, and PAL on the MetS risk.

**Results:**

A total of 591 incident MetS cases were diagnosed during 8.9 years of follow-up. The median total snack foods intake was 5.2 serving/week (IQR: 3.0–9.1). Total snack foods intake was positively associated with the MetS risk after adjusting for potential confounders (adjusted for age and gender, energy intake, total fiber intake, smoking status, PAL, education levels, family history of diabetes, family history of CVD events, and BMI). After adjustment for confounders, among snacks’ subgroups, candies and chocolate intake was positively associated with MetS risk. Moreover, among lower-educated and non-employed participants, candies and chocolate intake was positively associated with the MetS risk, by 38 and 43% respectively. Stratification based on PAL resulted a positive association between intake of total snack foods and candies and chocolates and risk of MetS among participants with low PAL.

**Conclusion:**

Snack foods, especially candies and chocolate, increased the risk of MetS, among individuals with a low PAL.

**Supplementary Information:**

The online version contains supplementary material available at 10.1186/s12937-021-00728-y.

## Introduction

It is well established that metabolic syndrome (MetS), a cluster of interrelated metabolic abnormalities, can occur from early childhood to late adulthood [1]. The National Cholesterol Education Program Adult Treatment Panel III has stated MetS as a promoter of cardiovascular disease (CVD) risk factors, which subsequently increases the risk of morbidity and all-cause mortality [2]. To reduce the burden of MetS, it is necessary to explore the effect of modifiable lifestyle factors, such as physical activity levels (PAL), that might have a preventing effect against the disease occurrence [3]. As indicated in several studies, the increasing trend of MetS is known to be associated with sedentary lifestyle behaviors, in addition to an ongoing nutrition transition, as a result of modernization that manifest itself through a higher intake of industrial and processed foods and is a major factor in the development of non-communicable diseases among populations [4, 5].

Snack foods intake has recently been emphasized as a potentially unhealthy eating habit [6]. Intake of energy-dense nutrient poor snacks from 1 serving per day to ≥ 2 serving per week, which are high in simple sugars, sodium, saturated and trans fatty acids, impose individuals to a higher risk of CVD and MetS [7]. Additionally, a 20% increase in the frequency of snacks intake was associated with weight gain and dysglycemia [8], and metabolically unhealthy obese subjects had a higher frequency of salty snacks, compared with metabolically healthy obese subjects [9]. Furthermore, snack foods was associated with imbalance energy intake and poor diet quality, which leads to weight gain as well as oxidative stress and inflammation [10, 11].

It is also well known that lifestyle factors alongside the diet may play an important role in the incidence of chronic diseases [3]. Socioeconomic status (SES), including education, occupation, and some lifestyle factors, such as PAL and smoking, are related to the MetS [12–14]. Two cross-sectional studies that have investigated the association between MetS and SES, measured by income and education, found that lower education levels as well as lower income were associated with MetS risk in both young and older adults [12, 13]. In another cross-sectional study, physical activity pattern was independently associated with the MetS in adults [14]. In a prospective study with 4 years of follow-up never smokers had lower risk of MetS compared to continual smokers among middle-aged adults [15].

To our knowledge, no prospective studies have considered SES and lifestyle factors (including education, occupation, PAL, and smoking) as mediators of the relation between snack foods intake and MetS risk. Because of that, the current study aimed to, firstly, investigate the association between snack foods intake and risk of MetS, and secondly, investigate the mediatory effect of SES and lifestyle factors on the association of snack foods and MetS, among adult population during 8.9 years of follow-up.

## Material and methods

### General characteristics

This study was conducted within the framework of the Tehran Lipid and Glucose Study (TLGS), a long-term prospective population-based study initiated in 1998 to determine the prevalence of non-communicable disease risk factors and its outcomes among the urban Tehranian population. The design and other details of TLGS were described elsewhere [16]. Briefly, the first survey phase initiated in 1999, with > 15,000 participants aged ≥ 3 years enrolling from district 13 of Tehran, the capital of Iran, using a multistage cluster random sampling method. Beginning in 1999 with 3-year intervals afterwards, participants were assessed for anthropometric measures, medication use, medical history of CVD risk factors, demographic, lifestyle factors and SES. To update all previous data, this information were documented every 3 years during face-to-face visits by the local research team. Phases II, III, IV, V, VI were prospective follow-up studies conducted between 2002–2005, 2006–2008, 2009–2011, 2012–2015, 2016–2018, respectively.

The current study used baseline examination data from phase III of the TLGS (2006–2008) and followed participants up to phase VI of TLGS (2016–2018), during an 8.91 (Interquartile Range: (IQR): 7.98–9.69) year follow-up. During the phase III of the TLGS, a representative sample of 4920 participants was randomly selected, for gathering their dietary information. From the 4920 selected participants in the present study, 3462 number of individuals, agreed to complete the FFQ. For the current study, 3265 adults aged 19–74 years with complete demographic, anthropometric, biochemical, and dietary data were selected. The characteristics of participants who completed the dietary assessment were similar to those of the total population in the phase III of TLGS [17]. The mean age and BMI of participants who completed the FFQ were 36.5 years and 25.6 kg/m^2^, compared with 35.4 years and 26.1 kg/m^2^ in participants who did not have nutritional data, in the phase III of the study. Among non-included participants, 32.9% had academic education and 21.5% were smokers, compared with 29.5 and 21.7% subjects who completed the FFQ.

**Fig. 1:**
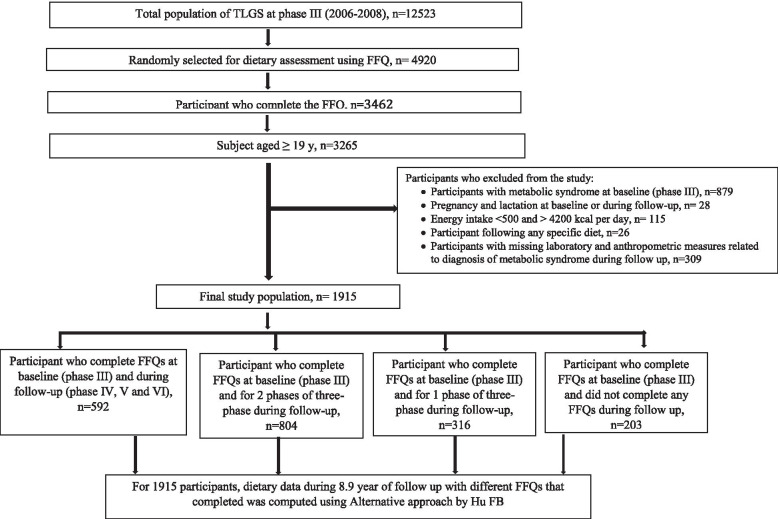
Flowchart of the study population, Tehran Lipid and Glucose Study (2006–2008 to 2016–2018)

We excluded those with MetS at baseline (*n* = 872), those who were pregnant or lactating at baseline and during follow-up (*n* = 28), those with under-or over-reporting of energy intakes (daily energy intake < 800 and > 4200 kcal per day) (*n* = 115), those were on any specific diets as result of diabetes, hypertension or hyperlipidemia (*n* = 26), and participants with missing biochemical and anthropometric measures related to diagnosis of MetS during follow-up (*n* = 309). Final analysis was conducted on 1915 participants until 2018 with a response rate of 66%, over the 8.9 (IQR: 7.98–9.69) year follow-up (Fig. 1). The study was approved by the research ethics committee of the Research Institute for Endocrine Sciences (RIES), Shahid Beheshti University of Medical Sciences, and written informed consent was obtained from the participants.

### Dietary assessment and snack foods intake

During face-to-face interviews, the regular dietary intakes of participants over the previous year were collected using a validated and reliable food frequency questionnaire (FFQ) [18–20]. Frequencies of consumption of each food item on a daily, weekly, or monthly basis during the previous year were documented according to a standard unit or portion size, evaluated in household measures specified for each food, during face-to-face interviews by trained dietitians, and consumed portion sizes were then converted to grams. The Iranian food composition table (FCT) was used to compute macro- and micronutrients [21]. For dietary exposures, long-term diet may be more important than the baseline measures. For this reason, we used the alternative approach that providing more weight to the recent diet, with reducing within subject variation. This approached was applied according to the Hu e.al formula [22]. Using this approach, the dietary intakes were calculated as the following: [2016–2018 diet / 2 + 2012–2015 diet / 4 + 2006–2008 diet / 8 + 2009–2011 diet / 8. Carried forward method was used to impute the missing dietary intake values [22]. From the initial number of 1915 participants at baseline, 592 participants completed all 4 FFQs (at bassline and during follow-up in phase IV, V, and VI), 804 participants completed 3 FFQs (at baseline and in two of the three phases of the follow up study), 316 participants completed 2 FFQs (at baseline and in one of the three phases of the follow up study), and 203 participants did not complete the any FFQs during follow-up (only at baseline). To impute missing values, last observation carried forward method was used.

Snack foods intake was collected using the FFQ reported a valid estimate against multiple dietary records. A good correlation coefficient was observed between FFQ and multiple 24 recalls (0.54 and 0.34 in males and females, respectively) and between two FFQs (0.42 and 0.53 in males and females, respectively [19]. Moreover, over the 8 year follow-up, there was reasonable reliability, validity and stability of the dietary patterns [18, 20].

In this study snack foods were divided into 1) biscuits and cakes, 2) candies and chocolate, 3) salty snacks. Biscuits and cakes included biscuits, cakes, cookies, crackers, confectionery, and traditional Iranian confectionaries including Gaz and Sohan. Candies and chocolates included chocolates, candies, cubed sugar, Noghl, and Nabat. Salty snacks consist of potato chips and puff (a corn snack or crisp coated with a mixture of cheese or cheese flavored). Total snack foods was calculated by the sum of them.

### Biochemical measurements

For assessing the biochemical measurements, after 12–14 h of overnight fasting, venous blood samples were obtained from all study participants placed into vacutainer tubes and centrifuged within 30–45 min of collection; fasting plasma glucose (FPG), high density lipoprotein cholesterol (HDL-C) and triglyceride (TG) analysis was done at the TLGS research laboratory on the day of sample collection, using a selector 2 auto-analyzer (Vital Scientific, Spankeren, the Netherlands) and commercial kits (Pars Azmoon Inc., Tehran, Iran). FPG was analyzed using an enzymatic colorimetric method with the glucose oxidase technique; inter- and intra-assay coefficients of variation (CV) at baseline and follow-up phases were both less than < 2.3%. HDL-C was analyzed after precipitation of apo-lipoprotein B-containing lipoproteins with phosphotungstic acid. TG was assayed using an enzymatic colorimetric method with glycerol phosphate oxidase. Both intra- and inter-assay CVs were below 3.0 and 2.1% for HDL-C and TG, respectively, in all baseline and follow-up assays.

### Socioeconomic status and lifestyle factors measurements

In terms of socioeconomic information, participants were divided into two groups according to their education levels. Subjects with a university degree were included in the higher-educated group and subjects with a degree less than a diploma were included in the lower-educated group. Moreover, based on economic status, participants were divided into two groups: 1) employed with income and 2) non-employed without income. The latest information on SES was used to classify individuals.

Participants were divided into groups of smokers or non-smokers. The smoker group included ex-smokers and current smoker’s participants and other participants were in the non-smoker group. PAL was assessed using the modifiable activity questionnaire (MAQ) that included a list of activities including leisure time, job, and household activities; the frequency and amount of time spent per week engaged in physical activity over the last year were recorded [23]. The validity and reliability the Persian version of the MAQ had been documented among adults [24]. PAL were expressed as metabolic equivalent hours per week (METs h/week) [25]. This value was used to categorize participants based on activities as low (< 3 MET hour-week) or medium/high (≥ 3 MET hour-week) PAL.

### Measurements of other variables

Throughout face-to-face interviews by trained interviewers, age, sex, and medical history data were gathered using a demographic questionnaire. Weight was recorded in minimally clothing to the nearest 0.1 kg on an SECA digital weighing scale (Seca 707; Seca Corporation, Hanover, Maryland; range 0.1–150 kg), and height was measured in a standing position, without shoes to the nearest 0.1 cm. Body mass index (BMI) was calculated as weight (kg) divided by the square of height (m2). Waist circumference (WC) was measured at the umbilical level using an un-stretched tape measure, and was recorded to the accuracy of 0.5 cm. After a 15 min resting, arterial blood pressure was measured manually, using a mercury sphygmomanometer with a suitable cuff size for each participant. Systolic blood pressure (SBP) was determined by the onset of the tapping Korotkoff sound, while diastolic blood pressure (DBP) was determined as the disappearance of the Korotkoff sound. Blood pressure was measured twice on the right arm twice at least 30 s apart; and the average was considered as the participant’s blood pressure.

According to the Joint Interim Statement, a MetS diagnosis requires the presence of three or more criteria [26] including 1.elevated glucose concentrations (FPG concentration ≥ 100 mg/dL or treatment with anti-hyperglycemic medications), 2.elevated serum TG concentration (≥ 150 mg/dL or treatment with anti-hypertriglyceridemia medications), 3.reduced serum HDL-C (< 50 mg/dL in women and < 40 mg/dL in men), 4.elevated blood pressure (≥ 130/85 mmHg or treatment with anti-hypertensive medications), and 5.enlarged abdominal circumference (≥ 95 cm according to the population- and country-specific cut-off points for Iranian adults of both genders) [27].

### Statistical analysis

All analyses were performed using SPSS software version 18 (SPSS, Chicago, IL, USA) and Stata software version 14.0 (StataCorp LLC, TX, USA). Normality of variables was tested by the histogram and Kolmogorov–Smirnov test. Quantitative variables were described as mean ± standard deviation (SD) or median (25th percentilee75th percentile), and qualitative variables were reported as percentage. In order to adjust energy, we used the residual method for all dietary factors [28]. The event date for MetS cases was described as the middle time between follow-up visit date at which MetS was detected for the first time, and the most recent follow-up visit, preceding the diagnosis; follow-up time was drawn from the difference between the calculated mid-time date and the date at which the subjects entered the study. For the censored and lost to follow-up subjects, survival time was the interval between the first and last observation dates.

Total snack foods intakes and its subgroup (serving/week) were modeled as tertiles. Baseline characteristics and dietary factors were stated across tertiles of total snack foods by general linear models and chi-square for continuous and categorical variables, respectively. Cox proportional hazards regression analyses were used to estimate the hazard ratios (HRs) and 95% confidence interval (CI) for incidence of MetS across tertiles of biscuits and cakes, candies and chocolates, salty snacks, and total snack foods. In the model 1, we used a univariate analysis. Model 2 was adjusted for age at baseline and gender. Model 3 was additionally adjusted for energy intake, total fiber intake, smoking status, PAL, education levels, family history of diabetes, and family history of CVD events. Finally, Model 4 was further adjusted for BMI at baseline. The proportional hazard assumption of the multivariable Cox model was assessed using Schoenfeld’s test of residuals (Supplementary Table 1). Linearity of trends were determined, using assigning the median values of the tertiles as continuous variables into the Cox regression models.

The effect modification of age, lifestyle factors and SES on the association between snack foods intake and incidence of MetS were evaluated using Cox regression. Based on PAL (MET hour-week), the participants were categorized into low PAL (< 3 MET hour-week) or medium/high (≥ 3 MET hour-week) PAL [29]. Participants were divided into groups of smokers or non-smokers. The Smoking group included ex-smokers and current smoker’s participants and other participants were in the non-smoker group. In terms of socioeconomic information, participants were divided into two groups according to their education levels. Subjects with a university degree were included in the higher-educated group and subjects with a degree less than a diploma were included in the lower-educated group. Moreover, participants were divided into two groups of full time employed and non-employed groups, as well. Finally, since the age range of the population study was very broad, participants were divided into two groups as aged 18–40 years and aged ≥ 41 years. Additionally, we evaluated the effect modification of age (18–40 years and ≥ 41 years) on the association between snack foods intake and risk of MetS based on lifestyle factors and SES categorization.

Stratified analysis based on lifestyle and SES factors were done when *P* < 0.20 [30]. Interaction was observed among candies and chocolate and SES and lifestyle factors as well as total snack foods intake and lifestyle factors (Supplementary Table 2). By multivariable Cox regression models, using joint classification, we further estimated the HRs and their 95% CIs for MetS according to PAL, smoking status, education levels, and occupation status with snack foods intake, during 8.9 years of follow-up. In all multivariable models, subjects with the snack foods intake lower than the median with low PAL, smokers, lower-educated, and non-employed were considered as reference. Data was adjusted for age, gender, smoking, PAL, education levels, occupational status, total energy intake, fiber intake, family history of diabetes, family history of CVD and BMI at baseline. In these modifier models, modifier variable was not adjusted. All statistical tests were considered statistically significant for *P*-values < 0.05.

## Results

We documented 591 new cases of MetS during the median follow-up of 8.9 years. The baseline mean (SD) age and BMI of the participants (male, 40.5%) was 36.5 (13.3) years and 25.6 (4.5), respectively. The median total snack foods intake was 5.2 serving/week (IQR: 3.0–9.1). From the total snack foods intake mentioned above, 2.2 (IQR: 1.2–3.8) was in form of cakes and biscuits, 1.6 serving (IQR: 0.95–2.4) in form of candies and chocolate and 1.7 serving/day (IQR: 0.6–3.8) in form of salty snacks.

Baseline characteristics of study participants are shown in Table 1. According to the results, by increasing the tertiles of snack foods intake, age, BMI at baseline, and employed participants decreased; however, smokers and participants with academic degrees increased significantly. Higher total snack foods were also positively associated with fasting plasma glucose, triglyceride concentrations, waist circumference, systolic and diastolic blood pressure at baseline. No significant changes were observed with regards to gender, PAL, family history of diabetes, and family history of CVD across tertiles of total snack foods intake.

**Table 1:** Baseline characteristics of study population across tertiles of total snack foods consumption

	**Total snack foods**			
	**T1**	**T3**	**T3**	***P* vlaue**
Participants (n)	639	637	639	
Age (year)	35.9 ± 0.5	34.1 ± 0.5	32.4 ± 0.5	0.014
Female (%)	55.9	60.3	62.4	0.051
Physical activity (MET hour-week)	5.1 ± 0.3	4.8 ± 0.3	4.9 ± 0.3	0.054
Smoker (%)	18.6	20.9	25.7	0.008
Academic degrees (%)	28.6	29.7	35.4	< 0.001
Employed (%)	47.3	44.4	37.7	0.002
Family history of diabetes (%)	29.1	34.9	33.1	0.114
Family history of CVD events (%)	18.5	17.6	18.8	0.900
BMI (kg/m^2^)	26.2 ± 0.2	25.5 ± 0.2	25.1 ± 0.2	< 0.001
Metabolic syndrome components				
Fasting plasma glucose (mg/dl)	96.3 ± 0.8	106.3 ± 0.8	128.0 ± 0.8	0.029
Triglyceride concentration (mg/dl)	108.3 ± 3.4	118.9 ± 3.4	126.7 ± 3.4	0.002
Systolic blood pressure (mmHg)	104.5 ± 0.8	118.5 ± 0.8	122.1 ± 0.8	0.002
Diastolic blood pressure (mmHg)	69.2 ± 0.6	71.6 ± 0.6	82.9 ± 0.6	0.005
Waist circumference (cm)	83.8 ± 0.8	85.2 ± 0.8	86.6 ± 0.8	0.035
HDL cholesterol (mg/dl)	45.7 ± 0.6	42.9 ± 0.6	44.6 ± 0.6	0.118

Values are mean ± SEM unless otherwise listed

*MET* Metabolic Equivalent, *BMI* Body mass index

Table 2 is shown the dietary intakes of study population according to the tertiles of total snack foods intake. Participants in the third tertile of total snack foods intake had higher energy, carbohydrate, and fat intake in comparison to those in the first tertile. Moreover, participants had higher saturated, and monounsaturated fatty acids (MUFA) intakes and lower poly-unsaturated fatty acids (PUFA). In comparison to the first tertile, participants in the third tertile of total snack foods intake consumed less fruits and dairy products, and higher biscuit and cakes, candies and chocolate, salty snacks, meat, processed meat and organ meat, and refined grains. Dietary Approach to Stop Hypertension (DASH diet) score, as an indicator of a healthy diet, reduced across tertiles of snack foods.

**Table 2:** Dietary intakes of study population across tertiles of total snack foods

	**Total snack foods**			
	**T1**	**T3**	**T3**	***P* value**
Total energy (Kcal/d)	2017 ± 33	2252 ± 33	2728 ± 33	< 0.001
Carbohydrate (% of total energy)	59.9 ± 0.4	61.6 ± 0.4	62.4 ± 0.4	0.001
Protein (% of total energy)	14.7 ± 0.3	14.6 ± 0.3	14.6 ± 0.3	0.965
Fat (% of total energy)	29.2 ± 0.2	30.0 ± 0.2	31.1 ± 0.2	< 0.001
SFA (% of total energy)	9.5 ± 0.1	9.9 ± 0.1	10.1 ± 0.1	< 0.001
MUFA (% of total energy)	10.0 ± 0.1	10.2 ± 0.1	10.5 ± 0.1	0.001
PUFA (% of total energy)	6.3 ± 0.1	6.0 ± 0.1	6.0 ± 0.1	0.002
Total fiber (g/d)	46.5 ± 0.7	40.8 ± 0.7	39.1 ± 0.7	< 0.001
Vegetables (g/d)	277 ± 6	290 ± 6	297 ± 6	0.081
Fruit (g/d)	429 ± 11	371 ± 11	349 ± 11	< 0.001
Meat, processed meat and organ meat (g/d)	20.5 ± 0.8	29.4 ± 0.8	34.2 ± 0.8	< 0.001
Whole grain (g/d)	136 ± 3.7	143 ± 3.7	146 ± 3.7	0.205
Refined grain (g/day)	299 ± 6.4	324 ± 6.4	372 ± 6.4	< 0.001
Dairy products (g/d)	442 ± 8	377 ± 8	350 ± 8	< 0.001
DASH score	19.3 ± 0.2	18.1 ± 0.2	17.4 ± 0.2	0.020
Total Snack foods	2.3 ± 0.2	5.5 ± 0.2	13.3 ± 0.2	< 0.001
Biscuit and cakes	0.9 ± 0.1	2.3 ± 0.1	5.4 ± 0.1	< 0.001
Candies and chocolate	0.5 ± 0.2	0.9 ± 0.2	1.7 ± 0.2	< 0.001
Salty snacks	0.9 ± .2	2.2 ± 0.2	6.1 ± 0.2	< 0.001

Values are mean ± SEM

The Vegetables include lettuce, tomatoes, cucumbers, mixed vegetables, squash, cooked leafy vegetables, eggplant, celery, green pea, green bean, carrot, garlic, onion, cruciferous vegetables (cauliflower, red and white cabbage), green pepper, spinach, turnip, green chilies, mushroom, pumpkin and potatoes

The Fruit include cantaloupe, melon, watermelon, pear, apricot, cherry, apple, peach, nectarine, green plum, fig, grapes, kiwi, grapefruit, orange, persimmon, tangerine, pomegranate, dates, prune (yellow and red), sour cherry, strawberry, banana, sweet lemon, lime lemon, cranberry, pineapple, raisins and dried fruits (fig, mulberry, peach and apricot)

The meat, processed meat and organ meat include red meats (beef, lamb), hamburger, sausage, bologna (beef), and organ meat (brain, tongue, feet, and head)

The whole grain include all whole and dark breads (barbari, sangak, taftoon, and toasted bread (whole grain)), popcorn, corn, cooked barley, bulgur and biscuits prepared with whole grains

Refined grain include white bread, baguette, cooked rice and pasta, cooked angel hair pasta, reshteh, and wheat flour

Dairy products include all kinds of milks (whole, low fat, skim, cocoa and chocolate), doogh (yogurt drink), yogurt (plain and whole), Yogurt (Concentrated and creamy), kashk, Cheese (plain and creamy)

*DASH* Dietary approach to stop hypertension

The association between total snack foods intake and its subgroups and risk of MetS are shown in Table 3. As results showed, by increasing the tertiles of biscuit and cakes intake, the risk of MetS increased significantly in the first model (HR: 1.46, 95% CI: 1.20–1.79); however, after adjustment for potential confounder in the second, third and fourth models, the association became null. Moreover, by increasing the tertile of candies and chocolate intake, the risk of MetS increased significantly in the first model (HR: 1.38, 95% CI: 1.12–1.69), the second (HR: 1.25, 95% CI: 1.01–1.47), the third (HR: 1.28, 95% CI: 1.03–1.58) and the fourth (HR: 1.28, 95% CI: 1.04–1.58) ones. In the first model of salty snacks intake, participants in the third tertile had a higher risk of MetS in comparison to the first tertile (HR: 1.74, 95% CI: 1.42–2.13); however, this relationship was not significant in the other models. Total snack foods intake was positively associated with the risk of MetS in the first (HR: 1.68, 95% CI: 1.37–2.05), the second (HR: 1.41, 95% CI: 1.12–1.81), the third (HR: 1.36, 95%CI: 1.08–1.71), and the fourth (HR: 1.32, 95% CI: 1.05–1.66) models.

**Table 3:** Multivariable adjusted hazard ratio (95% confidence interval) for metabolic syndrome across tertiles of total snack foods intake and its subgroups

	**Tertiles of intakes**			
	**T1**	**T2**	**T3**	***P* for trend**
**Metabolic syndrome**				
**Biscuit and cakes**				
Range of intake (serving/week)	≤ 1.5	1.6–3.1	≥ 3.2	
Median intake (serving/ week)	0.9	2.3	4.1	
Model 1	1	1.14 (0.93–1.41)	1.46 (1.20–1.79)	< 0.001
Model 2	1	1.09 (0.87–1.32)	1.17 (0.96–1.44)	0.284
Model 3	1	1.16 (0.94–1.44)	1.27 (1.02–1.59)	0.094
Model 4	1	1.12 (0.91–1.39)	1.23 (0.99–1.54)	0.168
**Candies and chocolate**				
Range of intake (serving/week)	≤ 0.5	0.6—1.7	≥ 1.8	
Median intake (serving/ week)	0.4	1.3	2.4	
Model 1	**1**	**1.33 (1.09–1.64)**	**1.38 (1.12–1.69)**	**0.004**
Model 2	**1**	**1.29 (1.05–1.58)**	**1.25 (1.01–1.47)**	**0.025**
Model 3	**1**	**1.34 (1.09–0.64)**	**1.28 (1.03–1.58)**	**0.015**
Model 4	**1**	**1.32 (1.08–1.63)**	**1.28 (1.04–1.58)**	**0.017**
**Salty snacks**				
Range of intake (serving/week)	≤ 0.9	1.0–2.8	≥ 2.9	
Median intake (serving/ week)	0.3	1.7	3.3	
Model 1	1	1.23 (1.00–1.53)	1.74 (1.42–2.13)	< 0.001
Model 2	1	1.04 (0.83–1.29)	1.01 (0.80–1.27)	0.926
Model 3	1	1.09 (0.87–1.35)	1.07 (0.85–1.36)	0.729
Model 4	1	0.98 (0.79–1.22)	1.01 (0.80–1.27)	0.969
**Total snack foods**				
Range of intake (serving/week)	≤ 3.6	3.7–5.4	≥ 5.5	
Median intake (serving/ week)	2.3	4.2	8.3	
Model 1	**1**	**1.21 (0.98–1.49)**	**1.68 (1.37–2.05)**	** < 0.001**
Model 2	**1**	**1.19 (0.95–1.50)**	**1.41 (1.12–1.81)**	**0.012**
Model 3	**1**	**1.23 (0.98–1.53)**	**1.36 (1.08–1.71)**	**0.030**
Model 4	**1**	**1.17 (0.94–1.46)**	**1.32 (1.05–1.66)**	**0.049**

Model 1 was crude

Model 2 was adjusted for age and gender

Model 3 was additionally adjusted for smoking, physical activity, education levels, occupation status, total energy intake, family history of diabetes, family history of cardiovascular disease (all variable that adjusted was at baseline)

Model 4 was additionally adjusted for BMI at baseline

In order to determine the modification effect of SES, lifestyle factors, and age groups on MetS risk across tertiles of different snack foods intake, stratification analysis was used. As illustrated in Table 4, when stratified by educational status, lower-educated participants in the second and third tertiles of candies and chocolate intake increased the risk of MetsS by 38% (HR: 1.38, 95%CI: 1.09–1.75) and 36% (HR: 1.36, 95%CI: 1.07–1.73) respectively, compared to the reference ones. In addition, risk of MetS increased in the third tertile of candies and chocolate intakes among non-employed (HR: 1.43, 95% CI: 1.05–1.93). Stratification based on PAL, resulted in the 37% (HR: 1.37, 95% CI: 1.02–1.84) increase of MetS risk across second tertile of biscuits and cakes intake, 42% (HR: 1.42, 95% CI: 1.06–1.90), and 46% (HR: 1.46, 95% CI: 1.08–1.96) increase of MetS risk across second and third tertiles of candies and chocolates intake, respectively in participants with low PAL. Moreover, the risk of MetS increased by 45% (HR: 1.45, 95% CI: 1.06–1.97) and 75% (HR: 1.75, 95% CI: 1.28–1.40) across second and third tertiles of total snack foods intake in participants with low PAL. No association was found between snack foods intake and risk of MetS in both smoker and non-smoker participants. Finally, by stratification based on age, the HR of MetS risk were 1.98 (95% CI: 1.34–2.91), 2.22 (1.70–2.91) and 2.72 (2.14–3.47) across first, second and third tertiles of biscuits and cakes intake, 2.59 (1.89–3.55), 3.31 (2.49–4.40), and 3.00 (2.29–3.94) across first, second and third tertiles of candies and chocolates intake, 1.78 (1.13–2.81), 1.95 (1.47–2.60), and 2.01 (1.59–2.54) across first, second and third tertiles of salty snacks, and 1.93 (1.17–3.17), 2.14 (1.62–2.82) and 2.89 (2.28–3.64) across first, second and third tertiles of total snacks food respectively in participants with age more than 41 years, compared with participant with the lowest intakes of snack foods and its subgroup intake and aged 18–40 year old.

**Table 4:** Multivariable adjusted hazard ratio (95% confidence interval) for metabolic syndrome across tertiles of snack foods intake among participants with different socioeconomic status, lifestyle factors, and age groups

	Lower-educated			Higher-educated		
	T1	T2	T3	T1	T2	T3
**Biscuit and cakes**	1	1.24 (0.97–1.58)	1.24 (097–1.59)	0.95 (0.68–1.33)	0.87 (0.59–1.28)	1.41 (0.92–2.07)
**Candies and chocolate**	1	**1.38 (1.09–1.75)**	1.36 (1.07–1.73)	1.05 (0.74–1.49)	1.20 (0.85–1.71)	1.05 (0.71–1.55)
**Salty snacks**	1	1.00 (0.78–1.29)	1.01 (0.78–1.31)	0.90 (0.63–1.30)	0.85 (0.60–1.20)	0.91 (0.63–1.31)
**Total snack foods**	1	1.22 (0.95–1.58)	1.27 (0.99–1.65)	0.92 (0.62–1.35)	0.91 (0.63–1.31)	1.32 (0.97–2.00)
	Non-employed			Employed		
	T1	T2	T3	T1	T2	T3
**Biscuit and cakes**	1	1.06 (0.79–1.42)	1.29 (0.95–1.75)	0.89 (0.64–1.25)	1.16 (0.85–1.59)	1.18 (0.87–1.60)
**Candies and chocolate**	1	1.33 (0.99–1.33)	**1.43 (1.05–1.93)**	1.05 (0.75–1.46)	**1.38 (1.01–1.89)**	1.23 (0.89–1.69)
**Salty snacks**	1	0.98 (0.72–1.33)	1.01 (0.73–1.37)	0.99 (0.71–1.41)	0.96 (0.68–1.35)	0.99 (0.73–1.38)
**Total snack foods**	1	1.05 (0.77–1.42)	1.35 (0.98–1.84)	0.90 (0.64–1.27)	1.18 (0.85–1.63)	1.12 (0.79–1.54)
	Smoker			Non-smoker		
	T1	T2	T3	T1	T2	T3
**Biscuit and cakes**	1	0.83 (0.53–1.29)	1.21 (0.81–1.82)	0.76 (0.54–1.08)	0.95 (0.67–1.34)	0.99 (0.70–1.40)
**Candies and chocolate**	1	1.42 (0.93–2.16)	1.25 (0.79–1.96)	0.90 (0.63–1.31)	1.19 (0.82–1.71)	1.17 (0.80–1.70)
**Salty snacks**	1	1.05 (0.69–1.59)	0.89 (0.57–1.39)	0.81 (0.59–1.15)	0.88 (0.62–1.25)	0.90 (0.63–1.28)
**Total snack foods**	1	1.20 (0.78–1.83)	1.14 (0.73–1.77)	0.81 (0.56–1.15)	0.99 (0.69–1.42)	1.14 (0.81–1.67)
	Low PAL			Medium and high PAL		
	T1	T2	T3	T1	T2	T3
**Biscuit and cakes**	1	1.37 (1.02–1.84)	1.33 (0.98–1.80)	0.91 (0.62–1.31)	0.93 (0.63–1.36)	0.93 (0.64–1.33)
**Candies and chocolate**	1	**1.42 (1.06–1.90)**	**1.46 (1.08–1.96)**	0.96 (0.66–1.40)	0.99 (0.68–1.44)	0.94 (0.65–1.36)
**Salty snacks**	1	0.99 (0.72–1.35)	1.09 (0.79–1.49)	0.79 (0.54–1.16)	0.75 (0.51–1.09)	0.77 (0.52–1.13)
**Total snack foods**	1	**1.45 (1.06–1.97)**	**1.75 (1.28–2.40)**	0.98 (0.67–1.44)	1.12 (0.75–1.67)	1.06 (0.73–1.54)
	Aged 18–40 year			Aged ≥ 41 year		
	T1	T2	T3	T1	T2	T3
**Biscuit and cakes**	1	0.94 (0.72–1.23)	0.94 (0.71–1.26)	**1.98 (1.34–2.91)**	**2.22 (1.70–2.91)**	**2.72 (2.14–3.47)**
**Candies and chocolate**	1	1.29 (0.98–1.69)	1.31 (0.98–1.74)	**2.59 (1.89–3.55)**	**3.31 (2.49–4.40)**	**3.00 (2.29–3.94)**
**Salty snacks**	1	0.93 (0.72–1.20)	1.01 (0.75–1.37)	**1.78 (1.13–2.81)**	**1.95 (1.47–2.60)**	**2.01 (1.59–2.54)**
**Total snack foods**	1	1.04 (0.81–1.35)	0.97 (0.71–131)	**1.93 (1.17–3.17)**	**2.14 (1.62–2.82)**	**2.89 (2.28–3.64)**

Data was adjusted for age, gender, smoking, physical activity, education levels, occupational status, total energy intake, fiber intake, family history of diabetes, family history of cardiovascular disease and BMI at baseline. In these modifier models, modifier variable was not adjusted

*PAL* Physical activity level

In addition, based on stratification by age, second and third tertiles of biscuit and cakes, candies and chocolate and total snack foods positively associated with risk of MetS among participants with aged ≥ 41 year old and low PAL (Supplementary Tables 3 and 4).

## Discussion

In this population-based prospective study, the association of different categories of snack foods intake with the risk of MetS, in addition to the modifiable effect of SES and lifestyle factors on this relationship have been evaluated. As the results show, after adjusting for potential confounders, by increasing the intake of chocolate and candies and total snack foods risk of MetS increased significantly. Moreover, after stratification of study population by their SES and lifestyle factors, the association between candies and chocolate intake and the risk of MetS was mediated by the education and PAL and the relationship between total snack foods intake and MetS was mediated only by PAL; so that the risk of MetS only increased in participants with low PAL. Finally, the association between all the snacks subtypes and the risk of MetS was mediated by the age groups.

The current study shows that unhealthy snack foods intake may be positively associated with the risk of MetS. Studies conducted to indicate the relationship between snack foods intake and risk of MetS are limited, their results are debatable, and most of them observed the association only among children and adolescents [10, 31, 32]. For example, our previous study investigated the effect of energy dense-nutrient poor snacks and incidence of MetS; no association was observed between biscuits and cakes, candies and chocolates and salty snacks with MetS among study population; however, in comparison to the current study the former was a cohort study with the average 3 years of follow-up period [31]. Moreover, a number of these studies have investigated the effect of snacks intake in the form of dietary patterns and as a holistic view [33, 34]. Woo et al. investigated the relation between three dietary patterns including traditional, meats, and snacks patterns with MetS in Korean adults. In this study no relation was observed between snacks and MetS and its components [33]. The author stated that the prevalence of MetS can be affected by the age; as by increasing the score of snack dietary pattern, mean age of study population decreased, although the snack foods intake in the current study was adversely related to age of study population. A systematic review and meta-analysis of observational studies indicated that prudent/healthy dietary patterns was indirectly and western/unhealthy dietary pattern was directly associated with the incidence of MetS [34]. It is worthy to state that in the Western/unhealthy dietary pattern, foods are most commonly consumed in form of cakes, cookies, soft drinks, red and processed meat, fast food, butter, and coffee. In the current study, by increasing the tertiles of total snack foods intake, the carbohydrate, total fat, SFA, and MUFA intakes increased and the PUFA intake decreased significantly and the unhealthy eating behaviors across the food groups including lower intakes of fruits and dairy products and higher intakes of red meat and refined grains was observed among study population. Kwon et al. stated that reduction of excessive carbohydrate intake with adequate fat intake, and taking into consideration optimal types of fat, are useful for prevention of MetS [35].

In the current study, the relation between MetS risk and snack foods as well as candies and chocolate intake was mediated by education and occupation status. Loucks et al. studied the SES of populations in early, middle, and late life period. In this study, income below the poverty line was associated with higher odds of MetS among women and similar findings were observed for educational status [12], this could be the results of better selected food items among participants with higher level of education [36], and food insecurity of low income participants which affects their food choices [37]. Although by increasing the intakes of total snack foods the smoker’s participants increased, no modifiable effect was observed between snack foods intake and MetS risk in non-smokers and smoker population. This emphasized the importance role of having a holistic view for studying the relation of diet-related disorders. Moreover, lower PAL modified the relation between candies and chocolate, and total snack foods intake with risk of MetS. According to Schaan et al. study, higher screen-based sedentary time is directly related to the MetS only among adolescents who consumed unhealthy snacks in front of screens [38]. Among the physiological systems that responds to PAL, one of the most demonstrable effects of PAL is its impact on insulin resistance [39]. Finally, as the risk of MetS have a positive relationship with the age of study population, in this study older population modified the relation between all the snacks subtypes and risk of MetS [40].

Including adults with a wide range of age, participants without previous history of CVD and diabetes, reliable and valid questionnaires for evaluation of dietary intakes and PAL, prospective design, long follow-up period, and adjustment for a large number of variables were some of the important strengths of the current study. Moreover, to our knowledge, this was the first study which assessed the mediatory effect of SES on the relation between snack foods intake and MetS risk across 8.9 years follow-up. Our study also had a number of limitations. One limitation to the generalizability of our findings is that it was necessary for us to exclude a number of participants with incomplete and invalid data on FFQ variables. Furthermore, the FFQ is limited to the number of snack foods that it can be captured and several popular snack products are not included. Moreover, we did not include data related to the liquid unhealthy snacks intake including sugar-sweetened beverages.

## Conclusion

It has become evident that there is a strong association between different snack foods and MetS risk factors, while SES and lifestyle factors may represent an important explanatory factor for this association. This study showed that education status and PAL may have an effect on the relationship between snack foods intake and MetS. Exposure throughout the life time may increase susceptibility to metabolic disorders, particularly in an environment of high unhealthy snacks intake, low PAL, and low SES. Identification of behavioral, as well as, cellular and molecular mechanisms behind the development of diet-related disorders will facilitate controlling the adverse consequences of chronic diseases among populations.

## Supplementary Information

**Additional file 1: Supplementary Table 1.** Proportional hazard assumption of the multivariable Cox model in snack foods and its subgroups. **Supplementary Table 2.** Interaction between consumption of total snack and its subgroups, lifestyle factors and socioeconomic status on the risk of MetS^*^. **Supplementary Table 3.** Multivariable adjusted hazard ratio (95% confidence interval) for metabolic syndrome across tertiles of snack foods intake among participants with aged < 40 year old. **Supplementary Table 4.** Multivariable adjusted hazard ratio (95% confidence interval) for metabolic syndrome across tertiles of snack foods intake among participants with aged ≥ 40 year old.

## Data Availability

All data generated or analyzed during this study are included in this published article.

## Abbreviations

BMI: Body mass index
CI: Confidence interval
CV: Coefficients of variation
CVD: Cardiovascular disease
DBP: Diastolic blood pressure
FCT: Food composition table
FFQ: Food frequency questionnaire
FPG: Fasting plasma glucose
HDL-C: High density lipoprotein cholesterol
HR: Hazard ratios
IQR: Interquartile Range
MAQ: Modifiable activity questionnaire
METs: Metabolic equivalent
MetS: Metabolic syndrome
MUFA: Monounsaturated fatty acids
PAL: Physical activity levels
PUFA: Polyunsaturated fatty acids
RIES: Research Institute for Endocrine Sciences
SBP: Systolic blood pressure
SD: Standard deviation
SES: Socioeconomic status
TG: Triglyceride
TLGS: Tehran Lipid and Glucose Study
USDA: US Department of Agriculture
WC: Waist circumference
