# Supplementary material for: Socioeconomic status and lifestyle factors modifies the association between snack foods intake and incidence of metabolic syndrome
Source: Nutr J. 2021 Jul 22;20:70. doi: 10.1186/s12937-021-00728-y (PMC8299611; doi:10.1186/s12937-021-00728-y)
Supplement: Supplementary file 1 — Additional file 1: Supplementary Table 1. Proportional hazard assumption of the multivariable Cox model in snack foods and its subgroups. Supplementary Table 2. Interaction between consumption of total snack and its subgroups, lifestyle factors and socioeconomic status on the risk of MetS*. Supplementary Table 3. Multivariable adjusted hazard ratio (95% confidence interval) for metabolic syndrome across tertiles of snack foods intake among participants with aged < 40 year old. Supplementary Table 4. Multivariable adjusted hazard ratio (95% confidence interval) for metabolic syndrome across tertiles of snack foods intake among participants with aged ≥ 40 year old. [file 12937_2021_728_MOESM1_ESM.docx]

| Supplementary Table 1. Proportional hazard assumption of the multivariable Cox model in snack foods and its subgroups | |
| --- | --- |
|  | P value |
| Biscuit and cakes | 0.148 |
| Candies and chocolate | 0.071 |
| Salty snacks | 0.142 |
| Total snack foods | 0.145 |

| Supplementary Table 2. Interaction between consumption of total snack and its subgroups, lifestyle factors and socioeconomic status on the risk of MetS ^*^ | | | | |
| --- | --- | --- | --- | --- |
|  | education | occupation | Smoking status | Physical activity levels |
| Biscuit and cakes | 0.341 | 0.375 | 0.294 | 0.777 |
| Candies and chocolate | 0.001 | 0.001 | 0.137 | 0.038 |
| Salty snakes | 0.547 | 0.295 | 0.539 | 0.458 |
| Total snacks | 0.312 | 0.785 | 0.393 | 0.029 |

**P value*

| Supplementary Table 3. Multivariable adjusted hazard ratio (95% confidence interval) for metabolic syndrome across tertiles of snack foods intake among participants with aged <40 year old | | | | | | |
| --- | --- | --- | --- | --- | --- | --- |
|  | Lower-educated | | | Higher-educated | | |
|  | T1 | T2 | T3 | T1 | T2 | T3 |
| **Biscuit and cakes** | 1 | 1.01 (0.72-1.43) | 0.93 (0.65-1.33) | 0.94 (0.55-1.61) | 0.78 (0.48-1.27) | 0.91 (0.58-1.41) |
| **Candies and chocolate** | 1 | 0.74 (0.53-1.04) | 0.78 (0.56-1.08) | 0.76 (0.47-1.23) | 0.80 (0.50-1.27) | 0.87 (0.57-1.32) |
| **Salty snacks** | 1 | 1.08 (0.77-1.53) | 0.89 (0.62-1.27) | 0.98 (0.63-1.52) | 0.65 (0.38-1.10) | 0.87 (0.54-4.41) |
| **Total snack foods** | 1 | 1.04 (0.74-1.46) | 0.88 (0.62-1.26) | 0.73 (0.43-1.23) | 0.99 (0.64-1.52) | 0.74 (0.46-1.19) |
|  | Non-employed | | | Employed | | |
|  | T1 | T2 | T3 | T1 | T2 | T3 |
| **Biscuit and cakes** | 1 | 1.27 (0.83-1.95) | 0.80 (0.49-1.29) | 0.91 (0.54-1.51) | 0.69 (0.41-1.17) | 0.96 (0.58-1.59) |
| **Candies and chocolate** | 1 | 0.73 (0.47-1.11) | 0.76 (0.49-1.14) | 0.71 (044-1.14) | 0.64 (0.40-1.04) | 0.69 (0.45-1.09) |
| **Salty snacks** | 1 | 1.17 (0.75-1.81) | 1.03 (0.64-1.68) | 0.87 (0.51-1.46) | 0.84 (0.51-1.41) | 1.00 (0.60-1.66) |
| **Total snack foods** | 1 | 1.12 (0.73-1.71) | 0.77 (0.47-1.24) | 0.82 (0.49-1.38) | 0.77 (0.47-1.28) | 0.84 (0.51-1.38) |
|  | Smoker | | | Non-smoker | | |
|  | T1 | T2 | T3 | T1 | T2 | T3 |
| **Biscuit and cakes** | 1 | 0.75 (0.43-1.30) | 0.87 (0.50-1.52) | 0.84 (0.52-1.36) | 0.91 (0.57-1.45) | 0.84 (0.52-1.35) |
| **Candies and chocolate** | 1 | 1.36 (0.77-2.40) | 1.11 (0.61-2.04) | 1.55 (0.93-2.57) | 1.03 (0.60-1.74) | 0.94 (0.55-1.74) |
| **Salty snacks** | 1 | 1.23 (0.67-2.27) | 1.71 (0.97-3.02) | 1.33 (0.80-2.21) | 1.36 (0.81-2.28) | 1.21 (0.71-2.06) |
| **Total snack foods** | 1 | 0.97 (0.54-1.72) | 1.05 (0.58-1.90) | 1.04 (0.62-1.73) | 1.08 (0.65-1.78) | 0.88 (0.52-1.50) |
|  | Low PAL | | | Medium and high PAL | | |
|  | T1 | T2 | T3 | T1 | T2 | T3 |
| **Biscuit and cakes** | 1 | 1.00 (0.67-1.49) | 0.87 (0.57-1.34) | 0.88 (0.58-1.34) | 0.79 (0.50-1.23) | 0.92 (0.60-1.40) |
| **Candies and chocolate** | 1 | 1.14 (0.54-1.37) | 1.19 (0.59-1.42) | 0.70 (0.46-1.06) | 0.73 (0.49-1.10) | 0.78 (0.54-1.15) |
| **Salty snacks** | 1 | 1.13 (0.75-1.69) | 1.05 (0.60-1.60) | 0.80 (0.50-1.26) | 0.83 (0.54-1.28) | 0.95 (0.60-1.48) |
| **Total snack foods** | 1 | 1.07 (0.73-1.58) | 0.79 (0.50-1.24) | 0.79 (0.50-1.24) | 0.73 (0.46-1.15) | 0.83 (0.54-1.29) |

| Supplementary Table 4. Multivariable adjusted hazard ratio (95% confidence interval) for metabolic syndrome across tertiles of snack foods intake among participants with aged ≥ 40 year old | | | | | | |
| --- | --- | --- | --- | --- | --- | --- |
|  | Lower-educated | | | Higher-educated | | |
|  | T1 | T2 | T3 | T1 | T2 | T3 |
| **Biscuit and cakes** | 1 | 0.95 (0.70-1.28) | 0.93 (0.67-1.29) | 0.92 (0.46-1.82) | 1.36 (0.81-2.29) | 0.37 (0.19-0.73) |
| **Candies and chocolate** | 1 | 0.99 (0.73-1.34) | 0.80 (0.58-1.10) | 0.91 (0.49-1.69) | 0.65 (0.36-1.16) | 0.66 (0.35-1.25) |
| **Salty snacks** | 1 | 1.29 (0.70-2.37) | 1.24 (0.68-2.25) | 1.18 (0.65-2.14) | 0.82 (0.36-1.88) | 1.06 (0.51-2.22) |
| **Total snack foods** | 1 | 0.97 (0.73-1.29) | 0.85 (0.61-1.18) | 0.98 (0.53-1.79) | 1.23 (0.72-2.12) | 0.33 (0.16-0.68) |
|  | Non-employed | | | Employed | | |
|  | T1 | T2 | T3 | T1 | T2 | T3 |
| **Biscuit and cakes** | 1 | 1.06 (0.75-1.49) | 0.87 (0.60-1.28) | 1.17 (0.70-1.94) | 1.11 (0.69-1.78) | 0.83 (0.51-1.37) |
| **Candies and chocolate** | 1 | 1.01 (0.71-1.44) | 0.86 (0.59-1.23) | 1.30 (0.79-2.12) | 1.03 (0.63-1.66) | 0.79 (0.48-1.30) |
| **Salty snacks** | 1 | 1.32 (0.92-1.88) | 1.06 (0.72-1.56) | 1.33 (0.94-2.67) | 0.89 (0.53-1.48) | 1.17 (0.72-1.88) |
| **Total snack foods** | 1 | 0.90 (0.64-1.28) | 0.86 (0.59-1.26) | 1.08 (0.66-1.76) | 1.22 (0.77-1.93) | 0.65 (0.39-1.08) |
|  | Smoker | | | Non-smoker | | |
|  | T1 | T2 | T3 | T1 | T2 | T3 |
| **Biscuit and cakes** | 1 | 1.36 (0.74-2.45) | 1.15 (0.62-2.12) | 1.48 (0.85-2.57) | 1.30 (0.92-1.82) | 1.36 (0.98-1.89) |
| **Candies and chocolate** | 1 | 1.02 (0.48-2.15) | 0.91 (0.45-1.83) | 1.31 (0.95-1.80) | 1.26 (0.91-1.74) | 1.28 (0.93-1.78) |
| **Salty snacks** | 1 | 1.10 (0.80-1.52) | 1.10 (0.79-1.53) | 0.96 (0.44-2.10) | 1.20 (0.59-2.45) | 1.18 (0.54-2.34) |
| **Total snack foods** | 1 | 1.09 (0.50-2.07) | 1.09 (0.50-2.09) | 1.08 (0.51-2.07) | 1.18 (0.61-2.48) | 1.17 (0.60-2.45) |
|  | Low PAL | | | Medium and high PAL | | |
|  | T1 | T2 | T3 | T1 | T2 | T3 |
| **Biscuit and cakes** | 1 | **1.81 (1.16-2.83)** | **1.73 (1.10-2.73)** | 1.16 (0.71-1.90) | 1.17 (0.75-1.81) | 1.17 (0.77-1.78) |
| **Candies and chocolate** | 1 | **1.58 (0.95-2.53)** | **1.90 (1.21-2.99)** | 1.26 (0.79-2.00) | 1.10 (0.71-1.69) | 1.18 (0.77-1.81) |
| **Salty snacks** | 1 | 1.42 (0.92-2.20) | 1.45 (0.96-2.20) | 1.38 (0.88-2.15) | 1.04 (0.68-1.57) | 0.96 (0.63-1.47) |
| **Total snack foods** | 1 | **1.70 (1.08-2.68)** | **1.70 (1.08-2.68)** | 1.24 (0.77-2.00) | 1.13 (0.73-1.75) | 1.15 (0.75-1.75) |
